# The Value of Applying Big Data Analytics in Health Supply Chain Management

**DOI:** 10.12688/f1000research.156525.1

**Published:** 2024-10-16

**Authors:** Dina Al Nuaimi, Niyi Awofeso

**Affiliations:** 1 Health and Environmental Studies, Hamdan Bin Mohammed Smart University, Dubai, Dubai, 0000, United Arab Emirates

**Keywords:** Supply chain management, OR, Healthcare supply chain management, OR, Healthcare supply chain management performance, Big data analytics, OR, Analytics, Enablers, OR, Success factors, ADVANCED MANUFACTURING TECHNOLOGY, AGILITY, ANALYTIC NETWORK PROCESS, ANALYTICS, AGILE MANUFACTURING

## Abstract

The use of big data analytics (BDAs) allows for the collection, management, and analysis of large volumes of data, which helps in making real-time decisions. This study aims to assess how the application of BDA impacts the performance of healthcare supply chain management (HCSCMP). Through a systematic literature review, the research explores how BDA enhances efficiency within healthcare supply chains (HCSCs) and identifies the critical factors enabling successful BDA implementation. A comprehensive search strategy was employed to analyze 65 papers, resulting in the inclusion of 39 studies published between 2016 and 2023. The review revealed a preference for literature reviews and questionnaires as the primary research methods. The findings indicate that BDA significantly improves HCSCs' efficiency, particularly in real-time decision-making and operational management. However, successful BDA implementation depends on addressing critical enablers and overcoming associated challenges.

## Introduction

“ *Data-driven decision-making is the backbone of innovation in healthcare supply chains, enhancing efficiency and patient outcomes.*”— Dina Al Nuaimi.

The study began with a descriptive analysis of academic research on BDA in the context of SCM, followed by a content analysis to assess the impact of BDA-based management systems on HCSCMP and to identify the key enablers and challenges for implementing BDA in HCSC.

Big Data Analytics (BDA) in healthcare is transformative, enabling the analysis of large datasets, identifying patterns, and developing predictive models through data mining techniques ( Ajah & Nweke, 2019; Batko & Ślęzak, 2022). The emergence of big data (BD) in supply chains (SC) has opened new avenues for enhancing efficiency and decision-making ( Nguyen et al., 2018; Hofmann & Rutschmann, 2018). BDA is crucial for managing, processing, and interpreting vast amounts of data, allowing organizations to derive actionable insights ( Tiwari, Wee, & Daryanto, 2018; Cozzoli et al., 2022). It integrates diverse data types, manages data quality, and provides comprehensive knowledge from massive datasets ( Ristevski & Chen, 2018; Zamani et al., 2022). While BDA is widely adopted in sectors such as education and healthcare ( Banu & Yakub, 2020; Galetsi et al., 2020), its application in healthcare supply chains (HCSC) is particularly critical, with the potential to significantly improve various aspects of healthcare, including Green Process Innovation ( Benzidia et al., 2023; Hasan et al., 2022). Healthcare facilities manage both structured and unstructured data. Structured data is easier to process due to its predefined schema, while unstructured data, which constitutes a large portion of BD, lacks clear structure and is challenging to analyze using traditional methods ( Al-Sai et al., 2022). Advanced BDA tools are increasingly capable of analyzing unstructured data; it is estimated that at least 60% of healthcare data is unstructured ( Awrahman et al., 2022). BD is characterized by its high volume, velocity, and variety, necessitating advanced technologies for effective management ( Lee et al., 2023). BDA provides the necessary tools to extract, store, analyze, and transform BD into valuable insights, supporting accurate decision-making and process optimization in HCSCs ( Ben Zineb et al., 2024).

Adopting BDA in HCSCs facilitates real-time service delivery, data-driven decision-making, and improved performance ( Araz et al., 2020). For example, it enhances inventory management (IM), improves demand forecasting accuracy, and facilitates information exchange, which are crucial for optimizing healthcare operations ( Batko & Ślęzak, 2022). However, improper data processing can lead to poor IM, inaccurate forecasts, and flawed decision-making ( Weng, 2022). Despite these benefits, research on BDA’s application in specific SCs like HCSCs is limited. Many organizations are in the early stages of BDA adoption due to a lack of understanding of BD management and its benefits ( Sarker, 2021). Further research is needed to explore how BDA can enhance healthcare supply chain management performance (HCSCMP) and to validate existing findings. Effective healthcare supply chain management (HCSCM) involves monitoring and optimizing production and distribution processes to improve efficiency in turning raw materials into final products and ensuring timely delivery to customers, thus maximizing value and providing a competitive advantage ( Investopedia, 2024; ASCM, 2023). This process typically includes five phases: planning, sourcing, manufacturing, delivery, and returns. In healthcare, SCM is crucial for ensuring the availability of medical products at the lowest possible cost, streamlining workflows, and optimizing IM. It also reduces losses from expired medicines and improves vendor management through digitalization ( Jabbarzadeh & Fahimnia, 2021).

This study aims to understand the value of applying BDA in HCSCM. The study focuses on direct observations and experiences with BDA, guided by the following research questions:

RQ1. What is the number of academic studies on Big Data Analytics in the context of Supply Chain Management, and what research methods and data collection techniques have been used in these studies?

RQ2. How does the application of Big Data Analytics enhance efficiency in Healthcare Supply Chains Management according to existing studies?

RQ3. What are the key enablers and challenges identified in the literature for the implementation of Big Data Analytics in Healthcare Supply Chains?

**Figure 1.**
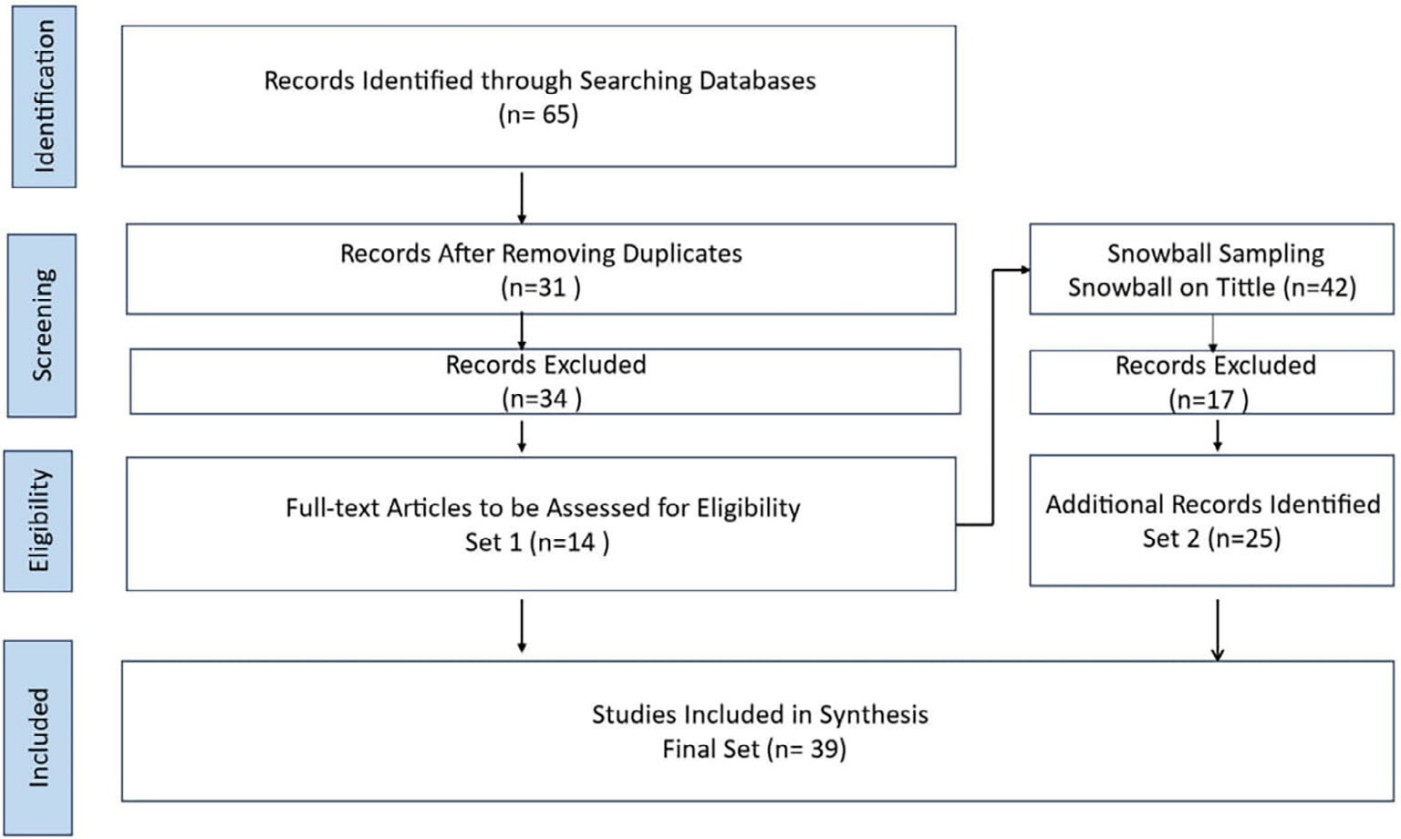
Systematic Literature Review Process.

The study centers on BDA in SCM, with related Systematic literature reviews (SLR). The SLR included screening 65 papers, ultimately including 39 papers from 2016 to 2023. The SLR highlights a preference for literature reviews and questionnaires. More longitudinal studies on BDA topics need to be conducted. The protocol for the current SLR, as presented in Figure 1, comprises three sequential processes: planning the review, performing the review, and presenting the review ( Behera, Bala, & Dhir, 2019; Tandon et al., 2020). The present SLR includes preset inclusion and exclusion criteria (see Figure 1), as recommended by prior literature (( Behera, Bala, & Dhir, 2019; Tandon et al., 2020; Khanra et al., 2020). This research novelty contributes to enhancing HCSCM by integrating BDA to improve efficiency, decision-making, and resilience. It enables a structured decision-making framework, tackles HCSCM challenges, and highlights factors for successful BDA implementation, which can help in effective deployment. The comparative analysis of BDA implementation in HCSC in different countries, including the United Arab Emirates (UAE), provides insights into global best practices and highlights the unique challenges and solutions in various contexts, offering a broader understanding of BDA's impact across different HCSCs.

### Big Data and Big Data analytics in Healthcare Supply Chain Management

The BD emerged in the 1990s to describe datasets that are too vast and complex for traditional IT systems to handle effectively ( Mallappallil et al., 2020). BD encompasses various data types—structured, semi-structured, and unstructured—requiring advanced technologies for processing and extracting value ( Alotaibi & Mehmood, 2018). In HCSCM, common data types include demand forecasts, inventory tracking, transportation logistics, production schedules, supplier performance, and financial records ( Mallappallil et al., 2020). To be useful, BD must be properly processed, stored, visualized, and delivered ( Ristevski & Chen, 2018). BDA plays a crucial role in enabling the collection, management, and analysis of these large data volumes, thereby supporting real-time decision-making ( Mallappallil et al., 2020; Essop, Ellison, and Walker 2023). Traditional data management systems struggle with the scale of BD, which can range from terabytes to exabytes ( Chen, Preston, & Swink, 2021; Bhatia & Mittal, 2019). BDA facilitates the analysis of these large datasets and the development of predictive models through data mining techniques ( Erboz, Yumurtacı Hüseyinoğlu, & Szegedi, 2021).

BDA refers to advanced tools that apply data mining and statistical analysis to create predictive analytics, enhancing strategic planning and operational efficiency ( Bagga & Chopra, 2018; Batko & Ślęzak, 2022). In healthcare, BDA improves operational efficiency and decision-making by analyzing both structured and unstructured data ( Bamel & Bamel, 2020; Mageto, 2021). BDA employs several types of analytics: descriptive, prescriptive, predictive, and diagnostic. Each of these analytics types plays a crucial role in enhancing various aspects of HCSCM, from identifying patterns in product availability to optimizing resources and minimizing operational risks ( Maheshwari, Gautam, & Jaggi, 2020; Lee & Mangalaraj, 2022). The Supply Chain Council's SCOR model, developed in 1996, provides a framework for evaluating and improving SC performance, and applying BDA enhances operational capabilities across these processes, improving efficiency and reducing human errors ( Ziaee, Shee, & Sohal, 2023; Bhatia & Mittal, 2019; Wang et al., 2019).

### Organizational Information Process Theory

The organizational information processing theory (OIPT) explains the organization’s capacity to interpret information meaningfully to enable informed decision-making ( Zhu et al., 2018). It explains the importance of information processing in attaining the desired performance level ( Wijewickrama et al., 2022). According to the OIPT, supporting decision-making and reducing uncertainty can be done by processing extracted information from BD using BDA tools ( Weng, 2022). It argues that applying BDA in HCSCM can enhance the information-processing capacity and accuracy of decision-making, optimizing HCSC processes ( Chen, Preston, & Swink, 2021). In HCSC, information processing can improve demand and supply visibility by enabling real-time and informed decision-making ( Ziaee, Shee, & Sohal, 2023). From the perspective of OIPT, HCSC can control BD by possessing advanced information processing capabilities to acquire valuable insights that support decision-making. Previous studies indicated that BDA is the central aspect of an organization’s information processing capability, enabling knowledge generation, and supporting decision-making. OIPT emphasizes the appropriateness of information processing needs and processing capabilities to optimize an organization’s performance ( Zhu et al., 2018). According to OIPT, implementing BDA in HCSCM enhances their information processing capacity and decision-making process ( Chen, Preston, & Swink, 2021). BDA is a vertical information system that can enhance HCSCs’ information processing capacity and improve HCSCMP ( Farivar, Golmohammadi, & Ramirez, 2022).

## Methods

The SLR was designed using a hybrid method ( Mourao et al., 2017). The hybrid method combines a keyword-based search, typical of a SLR, to define a start set and a snowball method ( Wohlin, 2014) to find relevant papers systematically. No recent SLRs focus specifically on the value of BDA in HCSCM. The SLR employed a keyword-based search to define an initial set of papers and used the snowball method ( Wohlin, 2014) to find additional relevant papers systematically. A standard keyword-based SLR ( Keele, 2007) can yield an extensive set of papers if the keywords are not restrictive enough or too small set if the keywords are overly restrictive. The search in Scopus in September 2023 included titles and abstracts, focusing on papers containing BDA and SCM keywords. The snowball approach ( Wohlin, 2014) is sensitive to the initial set of papers. The overall process is found in Figure 1. Four steps were conducted using the procedure suggested by the Preferred Reporting Items for Systematic Reviews and Meta-Analyses-Scoping Review (PRISMA-ScR) method. The four steps were

1. the determination of published papers,
2. the screening of the papers,
3. the selection of papers after assessment for eligibility, and
4. the inclusion of the selected papers for analysis.

This PRISMA diagram workflow illustrates the rigorous and systematic approach taken in the literature review process. The process from a broad search narrow down to high-quality studies most relevant to the research questions. Each step, from identification to inclusion, ensures the final synthesis is based on a thorough and methodologically sound literature review. The process begins with the identification phase, where relevant records are identified through comprehensive database searches. A systematic mapping study on empirical and systematic literature sets the stage for the SLR. In this case, 65 records were initially identified from various databases.

Once the records are gathered, the next step is to remove duplicates. This crucial step ensures that each study is considered only once, preventing any skewing of results due to repeated entries. After removing duplicates, 31 unique records remained for further screening. The screening phase involves a preliminary review of the titles and abstracts of the 31 records to determine their relevance to the research question. During this phase, 34 records were excluded based on predefined criteria such as language (non-English papers), study type (non-primary studies), and relevance (irrelevant topics). The remaining 14 full-text articles were assessed in detail in the eligibility phase to determine if they meet the inclusion criteria. The search was limited to original research articles written in full text in English and published between 2016 and 2023 in academic journals, ensuring the inclusion of recent and relevant studies. All the studies included in the review were peer-reviewed, ensuring their quality and reliability. This step ensures that only highly relevant and of sufficient quality studies are included in the final synthesis. After this detailed assessment, 34 articles were excluded for reasons such as being non-English papers (3 articles), not being primary studies (11 articles), and not being relevant to the research topic (20 articles).

Throughout the process, snowball sampling expanded the initial set by exploring relevant articles' references. This method ensures that all potentially relevant studies are considered, increasing the comprehensiveness of the review. The Snowballing step in the hybrid systematic review is snowballing the final start set papers. This method extended the start set by screening the references within the papers and those that cited them. Google Scholar was utilized to find forward references. The snowballed on the extended start set (14 papers) were done to get the final set of papers. In a snowball approach, both references in the paper (backward references) and papers referring to the paper (forward references) were screened ( Wohlin, 2014). The snowballing process was performed on titles only for backward and forward snowballing to ensure no relevant references were missing. This process was repeated until no new papers were found. Following screening and full reading, the final set consisted of 25 papers.

The final phase is the inclusion phase, where studies that passed the eligibility criteria are included in the synthesis. In this workflow, 39 studies were included in the final synthesis after additional snowball sampling. Snowball sampling involves reviewing the references of the included studies to identify any additional relevant studies, which added 25 more records to the initial set. Related SLRs and key publications were also considered to ensure a thorough and robust literature synthesis. The SLR process, as illustrated in the workflow, results in a final set of 39 studies that are included in the synthesis. These studies provide a comprehensive and reliable basis for understanding the research topic, ensuring that conclusions are based on a thorough and systematic examination of the existing literature. This detailed workflow ensures that the review process is transparent, reproducible, and methodologically sound, leading to high-quality and reliable research findings.

## Results analysis

In order to comprehend the extent of previous literature on BDA in SCM, we synthesized papers that provide an overview of BDA in SCM and identified them within reviews. This stage involves a thorough examination of these papers. This section presents the analysis results of the 39 selected peer-reviewed journal papers. The following sub-sections elaborate on the relevant findings. The study began with a descriptive analysis of academic research on BDA in the context of SCM, followed by a content analysis to assess the impact of BDA-based management systems on HCSCMP and to identify the key enablers and challenges for implementing BDA in HCSC.”

**RQ1. What is the number of academic studies on Big Data Analytics in the context of Supply Chain Management, and what research methods and data collection techniques have been used in these studies?**

### The number of academic studies on big data analytics in the context of supply chain management

**Figure 2.**
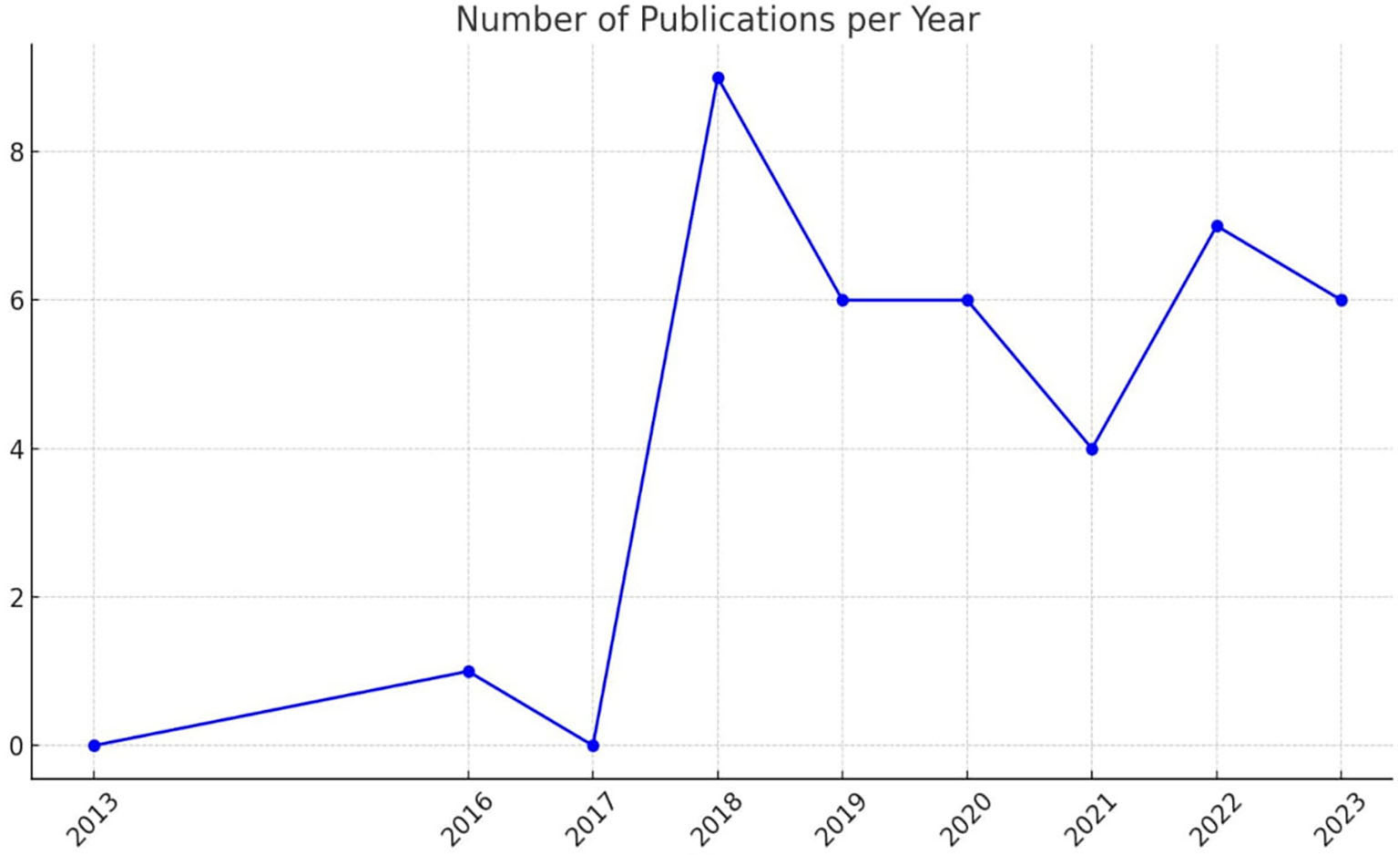
Number of Publications per Year.

Figure 2 illustrates the number of publications per year related to academic studies on BDA in the context of SCM.

The data indicates a significant increase in publications starting from 2018, with a peak that year. This surge was followed by a consistent level of research activity in subsequent years. Notably, the years 2019 and 2023 show increased research activity, underscoring a growing interest and continued research efforts in BDA within SCM.

**Figure 3.**
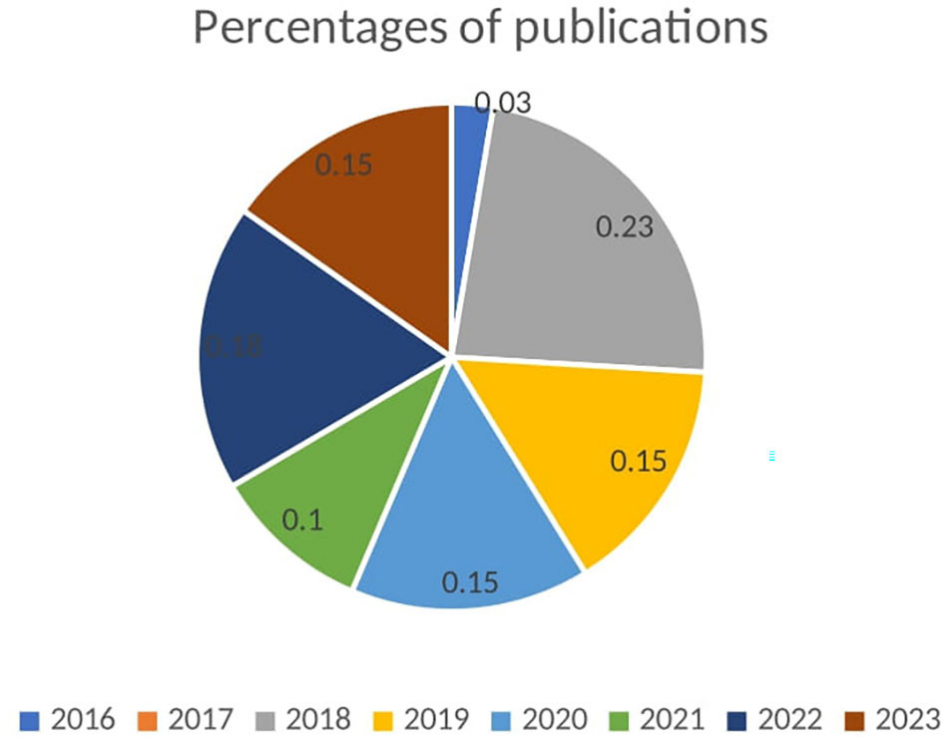
Percentages of Publications.

Figure 3 presents a pie chart depicting the percentages of publications per year. Each slice of the pie chart represents the proportion of publications for a specific year. The smallest slice corresponds to 2016, indicating a very modest research activity at 2.60%. The largest slice, representing 2018, shows a peak in research activity and interest in BDA within SCM, with 23% of the total publications. The years 2019 and 2020 each account for 15% of the publications, indicating stable and significant research activity during these years. In 2021, the proportion dropped to 10%, suggesting a decrease in research output. However, 2022 saw an increase to 18%, reflecting renewed interest and a rise in research efforts. The year 2023 returned to a stable level of research activity similar to 2019 and 2020, with 15% of the publications. The pie chart effectively conveys the fluctuations in research activity over the years. The peak in 2018 suggests a period of heightened interest or significant developments in the field of BDA within SCM. The stable proportions in 2019, 2020, and 2023 indicate consistent research output, while the increase in 2022 highlights renewed or sustained interest in this area.

### Research methods and data collection techniques have been used in big data analytics in the context of supply chain management studies

**Figure 4.**
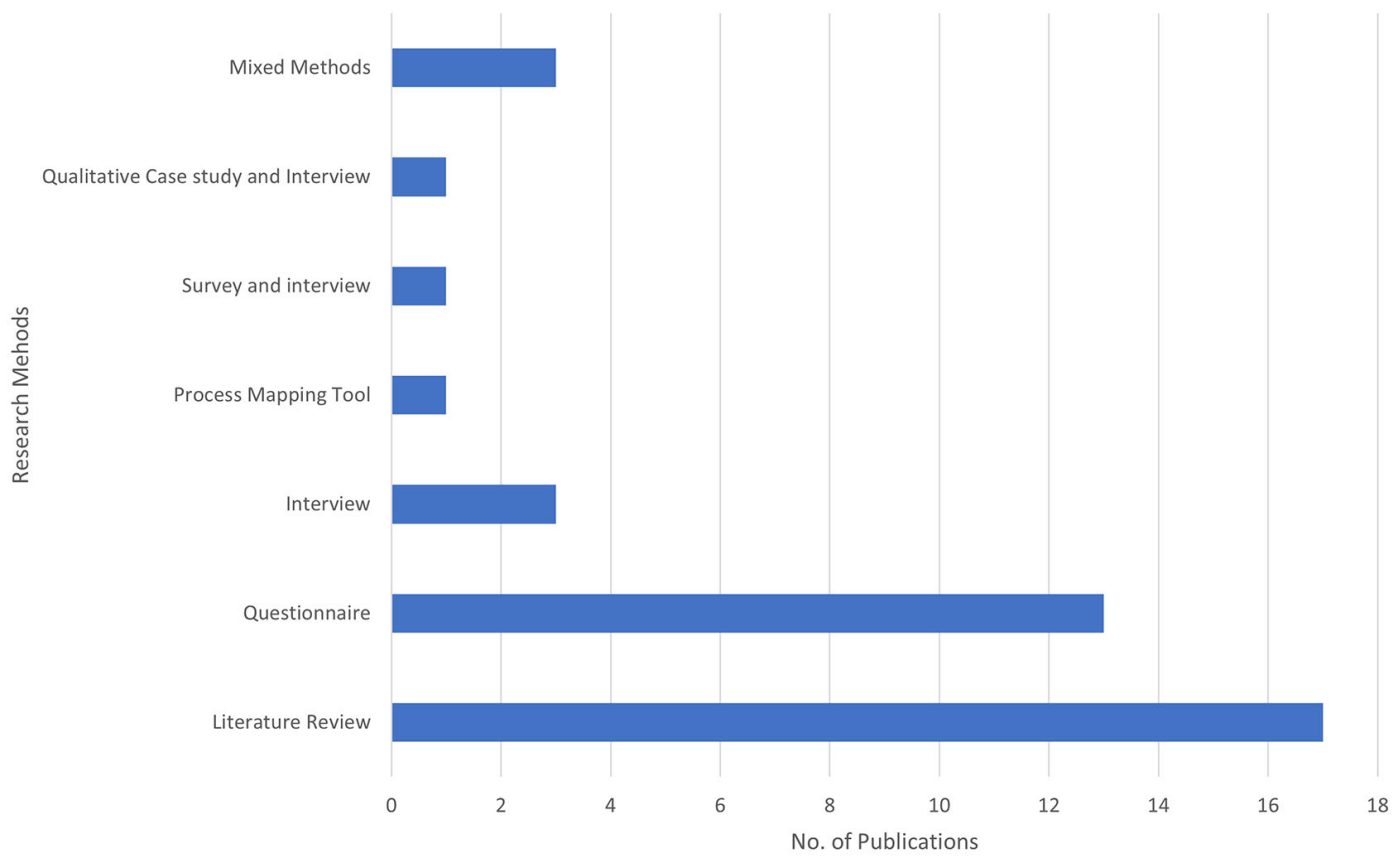
Number of Publications by Method Used.

The bar chart in Figure 4 illustrates the number of publications by different methods used in BDA research within the context of SCM. Each bar represents a distinct research method and the total number of publications employing that method.

Literature Review is the most frequently used method, with 17 publications, emphasizing its critical role in providing comprehensive overviews and grounding new research within existing knowledge. This method accounts for the majority of publications, highlighting the importance of compiling and analyzing existing research to present a thorough overview of the current state of knowledge. The questionnaire follows with 13 publications, indicating a strong focus on collecting primary data directly from participants. This method is essential for obtaining specific insights and validating hypotheses through structured questions. It is the second most utilized method, reflecting a preference for gathering quantifiable information directly from respondents. Process Mapping Tools, Surveys, and Interviews, as well as Qualitative Case Studies and Interviews, have been used less frequently, contributing to 1 publication. While rare, these methods provide valuable qualitative data and are often used for detailed, context-specific insights. Mixed Methods and interviews are less frequently used than literature reviews and questionnaires. Three publications represent interviews. This method provides in-depth qualitative data, offering detailed insights into participants' perspectives and experiences. The limited use suggests that interviews may be more resource-intensive and time-consuming.

Three publications represent Mixed Methods. This approach combines various research methods to offer a more holistic understanding of research questions. Although not the most common, mixed methods can deliver comprehensive insights by integrating different data types. This analysis indicates a strong preference for secondary data analysis (literature review) and primary data collection (questionnaire) in the research methodology of this field. Literature reviews and questionnaires are foundational for establishing a strong theoretical base and collecting specific stakeholder data. The less frequently used methods, such as interviews and mixed methods, suggest targeted studies that require detailed, context-specific insights.

The study comprised 17 non-empirical papers and 22 empirical papers. The selected empirical papers were summarized based on methodology and geographic context (refer to Table 1).

**Table 1.** Summary Table of Empirical Papers.

NO	Study	Methods	Context
1	Ziaee, Shee, and Sohal (2023)	Interviews	Australia
2	Raman, S. *et al.* (2018)	Questionnaire	United States, Middle East, Europe, Asia, and Australia
3	Oncioiu, I. *et al.* (2019)	Questionnaire	Romania
4	Mubarik and Mohd Rasi (2019)	Questionnaire	Pakistan
5	Batko and Ślęzak (2022)	Questionnaire	Poland
6	Lamba and Singh (2018)	Interviews	India
7	Farivar, Golmohammadi and Ramirez (2022)	Questionnaire	North America
8	Chen, Preston and Swink (2021)	Questionnaire	North America
9	Zhu et al. (2018)	Questionnaire	Multiple Countries Asia/Europe/USA
10	Benabdellah et al. (2016)	Questionnaire	Morocco
11	Chen, Preston and Swink (2021)	Questionnaire	North America
12	Bag et al. (2021)	Questionnaire	South Africa
13	Bag et al. (2023)	Questionnaire	South Africa
14	Agrawal and Madaan (2023)	Questionnaire	India
15	Benzidia et al. (2023)	Questionnaire	France
16	Johnson, Robert, and Smith (2019)	Mixed Methods	United States
17	Williams and Brown (2020)	Surveys and structured interviews	United States
18	Thompson and Thompson (2020)	Case studies and interviews	United States
19	Smith and Johnson (2018)	Mixed Methods	United States
20	Lee and Harris (2019)	Mixed Methods	United States
21	Hussain et al. (2023)	Process mapping tool	United Arab Emirates
22	Bamel and Bamel (2020)	Interview	India

**Figure 5.**
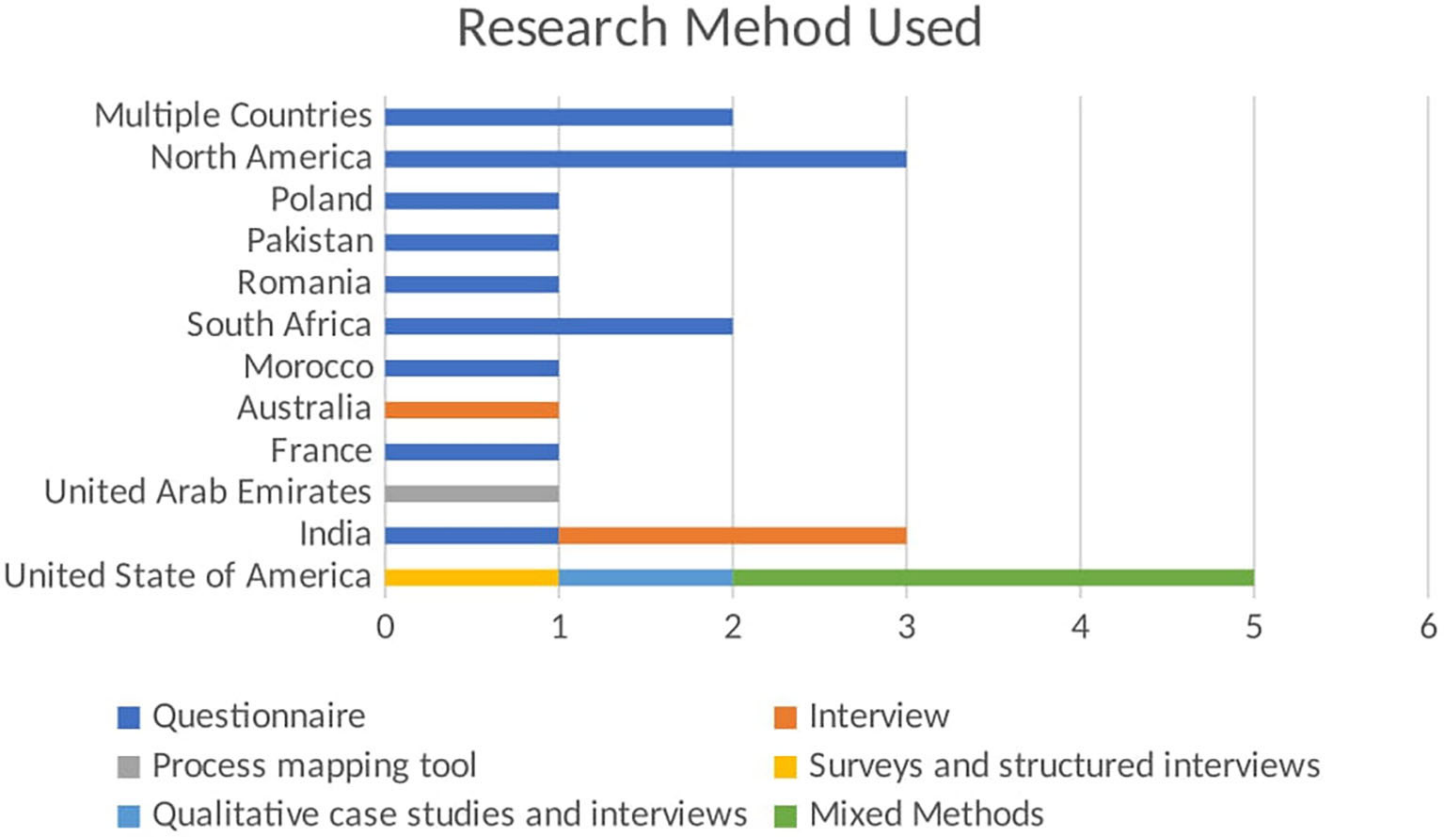
Empirical Research Method Used in Each Region.

The bar chart shown in Figure 5 visualizes the number of publications using different research methods across various regions. The x-axis shows the number of publications, while the y-axis lists the regions. Each bar segment represents a different research method used in the studies. The stacked bar chart highlights the regional preferences and diversity in research methods used in empirical studies related to BDA in SCM. Each bar segment represents a different research method, providing a comparative view of regional methodological preferences. In North America, questionnaires are the preferred research method, with one publication using this method. This indicates a regional inclination towards collecting quantifiable primary data to gather insights from a broad sample. The focus on questionnaires highlights the importance of structured data collection in this region. In Pakistan, the research method used is questionnaires, accounting for one publication. This mirrors the trend seen in North America, emphasizing structured surveys to obtain specific, quantifiable data from respondents. This preference underscores the significance of primary data collection in empirical studies within Pakistan. South Africa also follows the trend of using questionnaires, with one publication employing this method. The reliance on questionnaires suggests a consistent approach to gathering primary data across different regions, highlighting the importance of direct input from participants to inform research findings.

Australia distinguishes itself by using interviews, with one publication adopting this method. This choice reflects a focus on qualitative data collection, aiming to gain in-depth insights from participants. Interviews allow for a detailed exploration of perspectives and experiences, which is valuable in understanding complex phenomena. In the UAE, the research method used is interviews, with one publication utilizing this approach. Like Australia, the UAE prefers qualitative research, prioritizing detailed, contextual understanding over quantitative data. The United States of America demonstrates a diverse use of research methods:

- Surveys and Structured Interviews: 1 publication
- Qualitative Case Studies and Interviews: 1 publication
- Mixed Methods: 3 publications

This diversity indicates a comprehensive approach to empirical research, integrating various methodologies to cover quantitative and qualitative aspects. Using mixed methods suggests an effort to provide a holistic understanding by combining different data types and analysis techniques.

**RQ2. How does the application of big data analytics enhance efficiency in healthcare supply chains management according to existing studies?**

The next step of the study involves conducting a content analysis to illustrate the experiences of implementing BDA in SCM. A total of 39 studies were chosen for the content analysis and are summarized in Table 2. Table 2 provides a detailed assessment of the 39 systematic reviews. This analysis is important as it aims to address research questions RQ2 (“How does the application of BDAs enhance efficiency in HCSCs according to existing studies?”) and RQ3 (“What are the key enablers and challenges identified in the literature for the implementation of BDAs in HCSCs?”).

**Table 2.** Summary table of studies and its findings related to big data analytics in supply chain management.

No.	Study	Methods	The period of compilation of the data	Measured outcomes	Findings
1	Ziaee, Shee, & Sohal (2023)	Interviews	2023	Explore the benefits of BDA adoption in pharmaceutical supply chain	BDA capability is more helpful in HSC planning, delivery and return processes
2	Raman et al. (2018)	A survey was conducted among companies in the United States, the Middle East, Europe, Asia, and Australia	2018	Study impact of BDA on SCM	Results show that adopting BDA can affect the SCM's visibility and decrease the communication gap between demand and SCM
3	Oncioiu et al. (2019)	Quantitative study using a questionnaire	2019	Study the impact of BDA on company performance in SCM	Indicated that new capabilities and technologies, such as DBA, are required to manage and analyze information
4	Cozzoli et al. (2022)	Literature Review	2022	Examine the impact of BDA on healthcare management	Indicated the positive relationship between BDA and healthcare management
5	Mubarik & Mohd Rasi (2019)	Close-ended questionnaire	2019	Examine the impact of BDA on SC performance	Indicated a positive impact of BDA on planning, supplying, making, and IM
6	Dev et al. (2019)	Literature Review	2019	Study key performance indicators (KPIs) of SC with consideration of BDA	BDA providing real-time data processing and enhancing decision-making capabilities
7	Batko & Ślęzak (2022)	Questionnaire	2022	Examine impact of BDA in healthcare	BDA can support clinical decision-making
8	Bamel & Bamel (2020)	Interviews	2020	Determine BDA enablers of SC	BDA-based enablers are IT infrastructure for BDA; leadership commitment; staff skills for using BDA and financial support
9	Mageto (2021)	Literature Review	2021	Determine the relationship between BDA and Sustainable SCM	There is a strong relationship between BDA and Sustainable SCM
10	CİĞERCİ (2023)	Literature Review	2023	Study effects of BDA on SCM	BDA improve stock management, lowers costs, increases SC visibility
11	Hasan et al. (2022)	Systematic Literature Review	2022	Study the impact of BDA on SC operations	BDA can enhance the accuracy and timeliness of decision-making processes and optimize SC efficiency
12	Nguyen et al. (2018)	Literature Review	2018	Address the benefits of BDA in SCM	BDA can improve demand forecasting
13	Araz et al. (2020)	Literature Review	2020	Explore the role of BDA in risk management	Indicated that BDA enhances risk identification and decision-making capabilities
14	Hofmann & Rutschmann (2018)	Literature Review	2018	Explore role of BDA in improving forecasts’ accuracy	BDA enhances Forecasting Accuracy
15	Lamba & Singh, (2018)	Interview	2018	Determine and enablers the main for successful implementation of BDA in SCM	Indicates that top management commitment, financial support, technical skills, organizational structure and change management program are the main BDA enablers
16	Farivar, Golmohammadi & Ramirez (2022)	Survey	2022	Examine the role analytics capability and staff analytics skills in enhancing SC performance	Show that analytics capability must be accompanied by staff analytics skills to enhance SC performance
17	Tiwari, Wee, & Daryanto (2018)	Literature Review	2018	Explore impact of BDA in SCM	BDA enhances demand forecasting, decision-making, and inventory management
18	Zamani et al. (2022)	Literature Review	2022	Determine the role of BDA in SC resilience	BDA enhances SC resilience
19	Alotaibi & Mehmood (2018)	Literature Review	2018	Review the use of BDA in HCSC	BDA enhances decision-making in SC and increases transparency in HCSC
20	Chen, Preston & Swink (2021)	Questionnaire	2021	Investigates the impact of BDA in SCM	Application of BDA is related to better decision-making capability
21	Bhatia & Mittal (2019)	Literature Review	2019	Explore the application of BDA in HCSC	BDAs can enable timely and rapid healthcare service delivery
22	Maheshwari, Gautam & Jaggi, (2020)	Literature Review	2020	Explore the significance of BDA in SCM	BDA enhances demand forecasting accuracy, decreases inventory costs, optimized transportation routes, and improved risk management
23	Lee & Mangalaraj (2022)	Literature Review	2022	Explore effect of BDAs on SCM	BDA enhances visibility and resilience in SCM
24	Al-Sai et al. (2022)	Literature Review	2022	Explore impact of BDA	BDA enable real time decision making
25	Zhu et al. (2018)	Survey	2018	Examine role of BDA in supporting SC transparency	Analytics capability supports planning functions and impacts SC transparency
26	Benabdellah et al. (2016)	Survey	2016	Examine the role of BDA in SC	BDA enhances demand forecasting, visibility and transparency, and improves decision-making process in SCM
27	Chen, Preston & Swink (2021)	Questionnaire	2021	Investigates impact of BDA in decision-making in SCM	BDA can optimize SC by enhancing decision-making capability
28	Bag et al. (2021)	Questionnaire	2021	Examine the role of BDA in SC resilience	BDA can restore and increase SC resilience and improve decision making process
29	Bag et al. (2023)	Questionnaire	2023	Explores the effect of BDAs and AI (BDA-AI) technology-based in HCSC processes and performance	The BDA-AI platform will capacitate HCSC to deliver innovative performance
30	Hussain et al. (2023)	Process mapping tool, supplier-input-process-output-customer (SIPOC) chart	2023	Explores the challenges of BDA in HCSC	Determines numerous challenges in HCSC across the United Arab Emirates (UAE)
31	Agrawal & Madaan (2023)	Questionnaire	2023	Identifies barriers to BDA implementation in the HSC	Determines barriers to successful BDA implementation in the HSC
32	Benzidia et al. (2023)	Questionnaire	2023	Investigate the relationship between BDA in SCM and environmental and healthcare performance	The BDA affects environmental process integration to enhance environmental performance and healthcare performance
33	Johnson, Robert, & Smith (2019)	Mixed Methods	2019	Study the impact of BDA in inventory management	BDA improve inventory management efficiency
34	Martin & Lee (2018)	Literature Review	2018	Explore impacts of BDA in HCSC	Using BDA led to improvement in demand forecast accuracy Supportive Leadership and Organizational Culture are important enablers for BDA implementation Resistance to change can hinder the implementation of BDA
35	Lee et al. (2020)	Literature Review	2020	Explore impacts of BDA in Reducing Medical Supply Waste	BDA enables real-time decision-making
36	Williams & Brown (2020)	Surveys and structured interviews	2020	Explores the impact of BDA on order management	BDA led to reduction in order processing time and order errors Data quality and standardization issues are challenges for BDA implementation
37	Thompson & Thompson (2020)	Qualitative Case studies and interviews	2020	Explores Challenges in BDAs for Healthcare	BDA led to improvement in supplier reliability Regulatory Support and Compliance Frameworks are enablers for BDA implementation
38	Smith & Johnson (2018)	Mixed Methods	2018	Determines challenges and enablers for BDA implementation in HCSC	Advanced Technology Infrastructure is a critical enabler for the implementation of BDA in HCSC
39	Lee & Harris (2019)	Mixed Methods	2019	Examine the impact of skill development for BDA	Skill development important for BDA implementation High implementation costs for BDA included skilled personnel

BDA plays a transformative role in enhancing the efficiency HCSCM by optimizing various operational aspects such as IM, order management, demand forecasting, order fulfilment, and real-time decision-making. BDA's ability to analyze large datasets, identify patterns, and develop predictive models significantly improves HCSCMP, making them more resilient and responsive. One of the key contributions of BDA to HCSCM is IM. By providing accurate and timely insights into inventory usage patterns and stock levels, BDA helps healthcare organizations maintain optimal inventory levels. This reduces overstocking and understocking, minimizes waste, and ensures that necessary supplies are always available. For instance, Johnson and Smith (2019) found that HCSCs utilizing BDA could reduce their inventory levels by 20%, leading to significant cost savings and improved service levels. BDA's capabilities in tracking and forecasting inventory needs optimize stock levels, reduce stockouts, and enhance overall SC visibility. Similarly, Lee et al. (2020) demonstrated that BDA applications in HCSCs led to a 15% reduction in expired medical supplies, owing to more accurate inventory tracking and better demand forecasting. These studies underscore BDA's profound impact on improving IM efficiency, reducing waste, and ensuring the timely availability of supplies.

BDA also significantly improves efficiency in order management and demand forecasting. Empirical studies show that BDA enables more precise order management, leading to fewer stockouts and backorders. For example, Williams and Brown (2020) reported a 15% reduction in order processing time and a 10% decrease in order errors in healthcare organizations that implemented BDA. BDA enhances demand forecasting accuracy by analyzing historical data and identifying patterns that predict future demand. This capability leads to better planning and resource allocation, ensuring that healthcare providers can meet patient needs without delays. Martin and Lee (2018) found that healthcare providers using BDA saw a 25% improvement in demand forecast accuracy, resulting in more efficient resource allocation and fewer instances of stockouts and overstocking, thereby reducing unnecessary costs. CİĞERCİ (2023) further highlighted BDA's role in reducing uncertainties and enhancing responsiveness in SCM. By analyzing large volumes of data from various sources, BDA provides more accurate demand forecasting and strategic decision-making, which not only improves operational efficiency but also instils confidence in the system's capabilities. Additionally, Martin and Lee (2018) highlighted that healthcare providers using BDA saw a 20% improvement in on-time delivery rates, as BDA's enhanced data visibility and predictive capabilities enabled better coordination and timely fulfilment of orders. Raman et al. (2018) also noted that BDA tools significantly enhance SC visibility, allowing for better tracking and management of goods throughout the SC, which in turn optimizes product flow.

BDA's role in enhancing SC resilience is another critical aspect. CİĞERCİ (2023) found that BDA increases SC resilience by improving the ability to predict, respond to, and recover from disruptions. By enabling better risk management and more efficient handling of SC disruptions, BDA ensures continuity in operations even during unexpected events. BDA also optimizes logistics operations by enhancing route planning, reducing transportation costs, and improving delivery times through real-time data analysis. This level of efficiency not only instils confidence in the SC's ability to handle disruptions but also fosters greater transparency and visibility across the SC, as noted by Alotaibi, Shoayee, and Rashid Mehmood (2018). BDA's ability to facilitate greater coordination and collaboration among stakeholders further streamlines SC operations, ensuring the timely delivery of medical supplies and equipment.

Real-time decision-making is another area where BDA significantly enhances HCSCM efficiency. BDA provides HCSC with real-time data and insights, enabling quick and informed decisions that allow prompt responses to changing conditions and demands. Lee et al. (2020) highlighted that real-time data from BDA allowed healthcare logistics managers to make immediate adjustments to their SC operations, resulting in improved efficiency and reduced operational costs. Dev et al. (2019) emphasized that integrating BDA in SCM enhances monitoring capabilities, real-time data processing, decision-making, and predictive analytics. Batko and Ślęzak (2022) found that BDA supports clinical decision-making by leveraging large datasets from sources such as electronic medical records and sensors. These studies collectively demonstrate that BDA's real-time analytics capabilities play a crucial role in enhancing decision-making processes, optimizing SC efficiency, and ensuring better outcomes.

Furthermore, BDA contributes to improved SCM efficiency by enhancing supplier relationship management. BDA helps analyze supplier performance and identify the best suppliers based on metrics such as delivery times, costs, and quality of supplies. This leads to better supplier relationships and more reliable SCs. Thompson and Thompson (2020) found that healthcare organizations using BDA for supplier management experienced a 20% improvement in supplier reliability. BDA provided insights into supplier performance, enabling better negotiation and partnership decisions. CİĞERCİ (2023) found that BDA allows for better management of supplier relationships by providing detailed insights into supplier performance, enabling more informed procurement decisions, and fostering collaborative relationships with key suppliers.

BDA significantly enhances the efficiency of HCSCs by optimizing various operational aspects, including IM, order management, demand forecasting, order fulfillment, and real-time decision-making. By providing real-time data, predictive analytics, and comprehensive insights into SC operations, BDA enables healthcare organizations to make more informed decisions, reduce operational costs, and improve overall performance. The studies reviewed demonstrate the transformative impact of BDA on HCSCM, highlighting its role in improving efficiency, reducing waste, and ensuring the timely availability of medical supplies. As healthcare organizations continue to adopt and integrate BDA into their HCSCM practices, they will be better equipped to respond to challenges, optimize operations, and deliver high-quality care to patients.

**RQ3: What are the key enablers and challenges identified in the literature for the implementation of Big Data Analytics in healthcare supply chains?**

### Primary Enablers in Implementing Big Data Analytics in Healthcare Supply Chains

Lamba and Singh (2018) identified several critical enablers for successfully implementing BDA in SCM, including data quality, data governance, technological infrastructure, skilled personnel, and top management support. Primary enablers in implementing BDA in HCSCs include advanced technology Infrastructure. Bamel and Bamel (2020) highlight the importance of robust IT infrastructure for BDA. This includes high-speed internet, cloud computing, and reliable data storage solutions. Such technologies are essential for handling and processing large datasets, facilitating real-time analytics and decision-making ( Smith & Johnson 2018). Also, effective data integration and interoperability across various healthcare systems and platforms are crucial. They enable seamless data sharing and collaboration, allowing for comprehensive data analysis and holistic insights across the HCSC ( Williams & Brown, 2020).

Bamel and Bamel (2020) emphasize the need for a skilled workforce proficient in BDA tools and techniques. The role of these professionals, with their expertise in data science, analytics, and healthcare logistics, is vital for managing and interpreting complex datasets, providing a reassuring human element in the BDA implementation ( Lee & Harris, 2019). Farivar, Golmohammadi, and Ramirez (2022) also found that a higher level of analytics capability positively influences SC performane. Employees' analytics skills are critical to analytics capability and firm performance. Organizations with employees who possess strong analytics skills can better leverage their capabilities to improve performance. Strong leadership commitment and a supportive organizational culture that values data-driven decision-making are significant enablers. Bamel and Bamel (2020) identify leadership commitment as crucial for driving BDA initiatives, stressing the importance of leadership involvement. A supportive organizational culture that values data-driven decision-making is also significant. Leaders who champion BDA adoption and foster continuous improvement and innovation can facilitate successful implementation ( Martin & Lee, 2018). In addition to regulatory support and compliance frameworks. Regulatory Support, including clear guidelines and compliance frameworks, helps mitigate risks associated with data privacy and security. Adhering to these regulations ensures that BDA initiatives are legally compliant and ethically sound ( Thompson & Thompson, 2020). Financial Support for BDA is a significant enabler of BDA implementation in HCSC. Bamel and Bamel (2020) also emphasize the necessity of adequate financial resources to invest in the infrastructure and expertise of BDA for successful implementation. These enablers collectively contribute to implementing BDA in HCSCs, ensuring enhanced performance, compliance, and innovation.

### Primary Challenges in Implementing Big Data Analytics in Healthcare Supply Chains

Agrawal & Madaan (2023) identified several barriers to implementing BDA in HCSC. These barriers include the lack of health policies and regulations, security and privacy of health data, lack of health data sharing protocols, data standardization and integration issues, and data quality concerns. Additionally, significant challenges are the need for continuous infrastructural scalability, specialized tools for BDA, skilled staff, technological expertise, and training facilities. Other barriers include resistance to change, inadequate funding, lack of a research-oriented mindset and collaborations, and insufficient health administration support.

The challenges in implementing BDA in HCSC include data privacy and security concerns. Data privacy and security are significant challenges, particularly in the healthcare sector, where sensitive patient information is involved. Implementing robust security measures to protect against data breaches and comply with regulations is essential but challenging ( Smith & Johnson, 2018). The challenges also include data quality and standardization issues. Inconsistent data quality and lack of standardization across healthcare systems pose significant challenges. Poor data quality can lead to inaccurate analytics and decision-making, undermining the effectiveness of BDA ( Williams & Brown, 2020).

According to Lee and Harris (2019), high implementation costs for BDA are considered one of the main challenges. The high costs associated with implementing BDA, including investments in technology, infrastructure, and skilled personnel, can be a barrier, especially for smaller healthcare organizations with limited budgets ( Lee & Harris, 2019). In addition to organizational resistance to change. Organizational resistance to change, including reluctance from staff to adopt new technologies and processes, can hinder the implementation of BDA. Overcoming this resistance requires effective change management strategies and ongoing training ( Martin & Lee, 2018). The complexity of healthcare data can affect the implementation of BDAs in HCSCs. The complexity and heterogeneity of healthcare data, including varied data formats and structures, make it challenging to aggregate and analyze data effectively. Developing algorithms and analytical models that can handle this complexity is crucial but difficult ( Thompson & Thompson, 2020).

## Discussion

### Number of Academic Studies and Research Methods on Big Data Analytics in the Context of Supply Chain Management

The SLR results reveal a significant increase in academic studies on BDA in the context of SCM, a trend that started in 2018 and peaked that year. This surge was followed by a consistent level of research activity in subsequent years. Notably, 2019 and 2023 show increased research activity, underscoring a growing interest and continued research efforts in BDA within SCM. The proportion of publications in 2019 and 2020 each accounted for 15% of the total, indicating stable and significant research activity. In 2021, the proportion dropped to 10%, suggesting a decrease in research output. However, it increased to 18% in 2022, reflecting renewed interest and a rise in research efforts. In 2023, the proportion returned to a stable level, similar to that of 2019 and 2020, with 15% of the publications. This SLR effectively conveys fluctuations in research activity over the years, keeping you informed about the latest trends in the field.

The SLR results show a clear preference for secondary data analysis (literature review) and primary data collection (questionnaire) in the research methodology of this field. The study comprised 17 non-empirical papers and 22 empirical papers. Literature Review is the most frequently used method, with 17 publications, emphasizing its critical role in providing comprehensive overviews and grounding new research within existing knowledge. The questionnaire follows with 13 publications. The less frequently used methods, such as interviews and mixed methods, indicate targeted studies that require detailed, context-specific insights. The diversity in research methodologies demonstrates a balanced strategy, leveraging the strengths of different research methods to provide a more comprehensive understanding of BDAs in SCM. Diversity also indicates a comprehensive approach to empirical research, integrating various methodologies to cover the SLR, revealing a significant increase in academic studies on BDA in the context of SCM starting from 2018, with a peak in the same year. Subsequently, there has been a consistent level of research activity. Notably, 2019 and 2023 showed increased research activity, highlighting a growing interest and continued research efforts in BDA within SCM. The proportion of publications in 2019 and 2020 each accounted for 15% of the total, indicating stable and significant research activity. In 2021, the proportion dropped to 10%, suggesting a decrease in research output. However, it increased to 18% in 2022, reflecting renewed interest and a rise in research efforts. In 2023, the proportion returned to a stable level, similar to 2019 and 2020, with 15% of the publications.

### Efficiency Enhancements of Big Data Analytics in Healthcare Supply Chains

The research findings on BDA in HCSCs are significant. According to several studies, BDA plays a crucial role in enhancing efficiency in various HCSCM areas. BDA has been found to significantly improve IM by enabling healthcare organizations to maintain optimal inventory levels. For example, Johnson and Smith (2019) noted that hospitals using BDA were able to reduce their inventory by 20%, resulting in substantial cost savings and improved service levels. Similarly, Lee et al. (2020) demonstrated a 15% reduction in expired medical supplies by applying BDA in HCSCs. Moreover, Ziaee, Shee, and Sohal (2023) emphasized that BDA helps resolve drug shortages and optimize inventory through timely decision-making.

Empirical studies also indicate that BDA contributes to more precise order management and enhances demand forecasting accuracy. Williams and Brown (2020) reported a 15% reduction in order processing time and a 10% decrease in order errors. Additionally, Martin and Lee (2018) found that healthcare providers using BDA experienced a 25% improvement in demand forecast accuracy. CİĞERCİ (2023) highlighted BDA's role in reducing uncertainties and enhancing responsiveness in SCM, providing a sense of security and control in the face of potential challenges. Moreover, BDA significantly empowers healthcare professionals by improving order fulfilment processes. Martin and Lee (2018) highlighted a 20% improvement in on-time delivery rates. Raman et al. (2018) also found that BDA tools significantly enhance SC visibility, allowing better tracking and managing of goods and providing real-time data and insights for quick and informed decision-making. This empowerment instils a sense of confidence and capability in healthcare professionals.

Using BDA also helps analyze supplier performance and identify the best suppliers based on metrics such as delivery times, costs, and quality of supplies. Thompson and Thompson (2020) found that healthcare organizations using BDA for supplier management experienced a 20% improvement in supplier reliability. Additionally, CİĞERCİ (2023) found that BDA allows for better management of supplier relationships by providing detailed insights into supplier performance, fostering a sense of connection and collaboration with suppliers. Accurate medical product planning, demand forecasting, and replenishment can reduce inefficiencies in HCSCs. Applying BDA in HCSCs allows for qualitative and quantitative evaluation of suppliers' performance based on various factors. BDA-based systems collect precise data, allowing managers to make more accurate and prompt decisions based on predictive capabilities and real-time analytics.

BDA assists HCSC managers in decision-making and strategy implementation to enhance efficiency. The technology allows for analyzing patterns and unstructured data, providing predictive and traceability capabilities. It also enables informed decision-making regarding various operations such as demand planning, sourcing, procurement, manufacturing, inventory, matching demand and supply, and budget estimation. Furthermore, BDA regulates inventory levels, provides real-time inventory visibility across the SC, and aids in optimizing safety stock levels. Overall, BDA is crucial in improving HCSCM by facilitating real-time communication with partners, reducing costs, enhancing planning and implementation, and improving data collection and operational performance.

When comparing the implementation and impact of BDA in HCSCM across different nations, distinct patterns emerge. In Australia, the integration of BDA into HCSC processes has led to significant improvements in operational efficiency. Australian healthcare organizations have leveraged BDA to enhance IM, streamline procurement, and reduce wastage through better demand forecasting and real-time data analysis. This has resulted in more efficient HCSCs, capable of responding rapidly to changing demands and reducing operational costs ( Ziaee, Shee, & Sohal, 2023). Similarly, in the UAE, BDA infrastructure has been pivotal in improving HCSCM. The UAE's advanced BDA systems enable real-time risk management, optimized decision-making, and enhanced HCSC resilience. The ability to process large volumes of data swiftly and accurately allows UAE healthcare providers to maintain a robust HCSC, ensuring timely delivery of essential medical supplies and minimizing disruptions ( International Journal of Business Analytics and Security, 2023).

### Enablers and Challenges in Implementing Big Data Analytics in Healthcare Supply Chains

Lamba and Singh (2018) identified several critical enablers for successfully implementing BDA in SCM, including data quality, data governance, technological infrastructure, skilled personnel, and top management support. Bamel and Bamel (2020) highlight the importance of robust IT infrastructure, data integration, interoperability, a skilled workforce, and supportive leadership. Regulatory support and adequate financial resources are also critical enablers. BDA enhances data processing activities like mining, statistical, and predictive analysis. However, BDA’s effectiveness can only be attained by BDA enablers that enable HCSC capabilities and help HCSC achieve their optimal performance. BDA enablers include IT infrastructure for BDA, leadership commitment, staff skills for applying BDA, and financial support ( Bamel & Bamel, 2020).

The BD encompasses vast volumes of data that require meticulous analysis. The IT system plays a pivotal role in precise data analytics. It must be integrated into all HCSC processes to streamline medical products, information, and financial flows ( Lamba & Singh, 2018). BDA requires intelligent data processing software capable of collating data from diverse sources, continuously processing it in real-time into valuable information, and providing reports ( Mageto, 2021). Computing infrastructure supports HCSC by extracting information from unstructured data and enhancing SCM through stock optimization and cost reduction ( Bamel & Bamel, 2020). Leadership commitment is crucial for successfully implementing BDA in HCSC, providing a clear vision and support ( Lamba & Singh, 2018; Bamel & Bamel, 2020).

Effective data processing requires technical skills and trained personnel to analyze data and derive insights for better decision-making ( Mageto, 2021). Technical skills involve extracting value from BD through statistics and data science ( Gupta et al., 2019). Competent data scientists and staff are essential for data computing and analysis, maximizing the benefits of BDA technology ( Bamel & Bamel, 2020; Farivar, Golmohammadi, & Ramirez, 2022). HCSC staff must be skilled in qualitative and quantitative forecasting techniques and analytical methods ( Waller & Fawcett, 2013). Finally, implementing BDA requires substantial financial support for technologies, training, and acquiring data analytics expertise with high technical skills ( Lamba & Singh, 2018; Bamel & Bamel, 2020).

Agrawal and Madaan (2023) identify several barriers, including the lack of health policies and regulations, security and privacy of health data, lack of data sharing protocols, data standardization issues, high implementation costs, and organizational resistance to change. Ensuring data privacy and security, maintaining data quality, managing high implementation costs, and overcoming organizational resistance are significant challenges in implementing BDA in HCSCs. The SLR highlights the significant impact of BDA on HCSCs. BDA enhances efficiency by optimizing IM, improving order and demand forecasting, streamlining order fulfilment, enabling real-time decision-making, and improving supplier relationship management. The successful implementation of BDA relies on advanced technology infrastructure, data integration, a skilled workforce, supportive leadership, regulatory support, and financial resources. However, challenges such as lack of health policies and regulations, data privacy and security, data quality, high implementation costs, and organizational resistance must be addressed to realize the benefits of BDA in HCSCs fully.

Certain nations may encounter challenges when implementing BDA due to differences in technological infrastructure and data governance frameworks. For instance, research conducted in the UK has both highlighted the potential benefits and the obstacles in adopting BDA for SCM. While BDA can significantly improve operational performance, issues such as data privacy, the high cost of technology, and the need for specialized skills can hinder its full adoption ( Essop, Ellison, and Walker, 2023). The extent of BDA's impact in Europe or North America largely depends on the existing technological infrastructure, regulatory environment, and organizational readiness. In some cases, such as in the UAE and Australia, the benefits are more evident due to robust infrastructure and supportive governance. However, in other regions, these factors may limit the effectiveness of BDA ( Ziaee, Shee, & Sohal, 2023).

## Conclusion

The research trend in BDA for SCM shows increasing interest and sustained activity, particularly in the healthcare sector. This SLR highlights a preference for literature reviews and questionnaires, which establish strong theoretical bases and gather specific stakeholder data. Less frequent methods, like interviews and mixed methods, are used for detailed, context-specific insights, reflecting the diverse needs of empirical studies in BDA and SCM. BDA significantly enhances efficiency in HCSCs by optimizing IM, improving order management, refining demand forecasting, streamlining order fulfilment, and enabling real-time decision-making. These improvements lead to better resource allocation, cost savings, and service levels. BDA's real-time data and analytics capabilities enhance SC visibility, resilience, and logistics operations, making SCM more efficient and responsive. Successful implementation of BDA in HCSCs relies on critical enablers such as advanced technology infrastructure, data integration, a skilled workforce, supportive leadership, regulatory frameworks, and financial resources. However, challenges like data privacy and security, high implementation costs, and continuous staff training must be addressed to realize BDA's benefits fully. Addressing key enablers and overcoming challenges will significantly improve HCSCMP.

Developing robust frameworks and solutions to address data privacy and security challenges in BDA implementation is critical. It is crucial to develop strong frameworks and solutions to tackle data privacy and security challenges in BDA implementation. It is also essential to conduct detailed cost-benefit analyses to understand the financial implications of implementing BDA in HCSCs. Furthermore, it is important to investigate the effectiveness of various training and development programs designed to enhance the BDA skills of HCSC professionals. Similarly, examining the impact of changing regulatory frameworks on the implementation and effectiveness of BDA in HCSCM can provide deeper insights and practical solutions to enhance the adoption and effectiveness of BDA in HCSCs and beyond. Also, conducting detailed cost-benefit analyses is essential to better understand the financial implications of implementing BDA in HCSCs. Investigating the effectiveness of various training and development programs designed to enhance the BDA skills of HCSC professionals and examining the impact of changing regulatory frameworks on the implementation and effectiveness of BDA in HCSCM can provide deeper insights and practical solutions to enhance the adoption and effectiveness of BDA in HCSCs and beyond.

Similarly, researchers might consider diversifying their methodologies to include more mixed methods and qualitative approaches, which can provide richer, more nuanced insights. Conducting longitudinal studies to understand the long-term impacts of BDA on HCSCMP and identify trends over time is also recommended.

## Ethics and consent

Ethical approval and consent were not required

## Data Availability

No data are associated with this article. Open Science Framework (OSF): The value of applying big data analytics in health supply chain management, https://doi.org/10.17605/OSF.IO/ZGSCU ( Al Nuaimi & Al Nuaimi, 2024). This project contains the following extended data:
• Empirical Research Method Used in Each Region-1.jpg • Number of Publications by Method Used-1.jpg • Number of Publications per Year-1.jpg • Percentages of Publications-1.jpg •
PRISMA_2020_checklist and workflow - BDA Value.pdf • Summary Table of Empirical Papers.docx • Summary Table of Studies and Its Findings Related to Big Data Analytics in Supply Chain Management.docx • Systematic Literature Review Process-1.jpg • Systematic Literature Review Process.docx •
Table 1. Summary Table of Empirical Papers.xlsx •
Table 2. Summary Table of Studies and Its Findings Related to Big Data Analytics in Supply Chain Management Data is available under the terms of the *CC0 1.0 Universal.* Open Science Framework (OSF) Repository: PRISMA checklist and flow chart for ‘The value of applying big data analytics in health supply chain management’, https://doi.org/10.17605/OSF.IO/ZGSCU ( Al Nuaimi & Al Nuaimi, 2024). Data are available under the terms of the *CC0 1.0 Universal*
