# Supplementary material for: The Value of Applying Big Data Analytics in Health Supply Chain Management
Source: F1000Res. 2025 Feb 19;13:1237. Originally published 2024 Oct 16. [Version 4] doi: 10.12688/f1000research.156525.4 (PMC11842960; doi:10.12688/f1000research.156525.4)
Supplement: Peer review history

# Reviewer report: Reviewer response for version 4

**DOI:** 10.5256/f1000research.178092.r367582

**Published:** 2025-02-20

**Authors:** Martin Beaulieu

**Affiliations:** 1 HEC Montréal, Montreal, Canada

Sorry I missed the previous revisions of your paper. I find this version much improved and see no further changes to suggest.

Are the rationale for, and objectives of, the Systematic Review clearly stated?

Yes

Is the statistical analysis and its interpretation appropriate?

Partly

If this is a Living Systematic Review, is the ‘living’ method appropriate and is the search schedule clearly defined and justified? (‘Living Systematic Review’ or a variation of this term should be included in the title.)

Yes

Are sufficient details of the methods and analysis provided to allow replication by others?

Partly

Are the conclusions drawn adequately supported by the results presented in the review?

Partly

Reviewer Expertise:

NA

I confirm that I have read this submission and believe that I have an appropriate level of expertise to confirm that it is of an acceptable scientific standard.

# Reviewer report: Reviewer response for version 3

**DOI:** 10.5256/f1000research.175869.r349213

**Published:** 2024-12-20

**Authors:** Abdüssamet Polater

**Affiliations:** 1 Erzincan Binali Yildirim Universitesi, Erzincan, Erzincan, Turkey

The paper is scientifically sound in its current form.

Are the rationale for, and objectives of, the Systematic Review clearly stated?

Partly

Is the statistical analysis and its interpretation appropriate?

Partly

If this is a Living Systematic Review, is the ‘living’ method appropriate and is the search schedule clearly defined and justified? (‘Living Systematic Review’ or a variation of this term should be included in the title.)

Yes

Are sufficient details of the methods and analysis provided to allow replication by others?

Partly

Are the conclusions drawn adequately supported by the results presented in the review?

Yes

Reviewer Expertise:

Supply chain management; Logistics management

I confirm that I have read this submission and believe that I have an appropriate level of expertise to confirm that it is of an acceptable scientific standard.

# Reviewer report: Reviewer response for version 2

**DOI:** 10.5256/f1000research.174834.r343643

**Published:** 2024-12-06

**Authors:** Abdüssamet Polater

**Affiliations:** 1 Erzincan Binali Yildirim Universitesi, Erzincan, Erzincan, Turkey

The paper is scientifically sound in its current form and only minor, if any, improvements are suggested. However, I only require from the authors to include the explanations that they made in the response letter into the manuscript's methodology section. They should include the reason of starting to screen in 2016, the keywords used for screening, journals (science category, index type etc.) were included and how they analyzed the papers included.

Are the rationale for, and objectives of, the Systematic Review clearly stated?

Partly

Is the statistical analysis and its interpretation appropriate?

Partly

If this is a Living Systematic Review, is the ‘living’ method appropriate and is the search schedule clearly defined and justified? (‘Living Systematic Review’ or a variation of this term should be included in the title.)

Yes

Are sufficient details of the methods and analysis provided to allow replication by others?

Partly

Are the conclusions drawn adequately supported by the results presented in the review?

Yes

Reviewer Expertise:

Supply chain management; Logistics management

I confirm that I have read this submission and believe that I have an appropriate level of expertise to confirm that it is of an acceptable scientific standard, however I have significant reservations, as outlined above.

# Response

**Published:** 2024-12-10

**Authors:** دينا النعيمي

Thank you for your positive feedback and for recognizing the scientific merit of our paper. We greatly appreciate your constructive suggestion to include additional details in the Methodology section.

 In response, we have incorporated the following explanations into the manuscript:

 The rationale for starting the screening process in 2016.

 The specific keywords used for the screening process.

 The journal inclusion criteria (e.g., science category, index type).

 The analytical approach was applied to the selected papers.

 These details have been added to the Methodology section. We believe these revisions enhance the clarity and transparency of our study.

 Thank you for your thoughtful input, which has helped us improve our manuscript.

# Reviewer report: Reviewer response for version 1

**DOI:** 10.5256/f1000research.171850.r333492

**Published:** 2024-11-12

**Authors:** Abdüssamet Polater

**Affiliations:** 1 Erzincan Binali Yildirim Universitesi, Erzincan, Erzincan, Turkey

**Comments for the Abstract**

 The aim of the study is confusing in the abstract section. It is stated that “This study aims to assess how the application of BDA impacts the performance of healthcare supply chain management (HCSCMP).” Then, investigating the efficiency and success factors are stated as an aim of the study.

**Comments for the Introduction**

 “The study began with a descriptive analysis of academic research on BDA in the context of SCM, followed by a content analysis to assess the impact of BDA-based management systems on HCSCMP and to identify the key enablers and challenges for implementing BDA in HCSC.” The location of this sentence can be changed in the introduction sentence.

 The paragraph which starts with “Big Data Analytics (BDA) in healthcare is transformative…” is fine until the “Healthcare facilities manage both structured and unstructured data.” sentence. Then suddenly the authors starts to talk about the “structured and unstructured data”. Is there any need to talk about “structured and unstructured data” here?

 The paragraph starting with the “Adopting BDA in HCSCs facilitates real-time service delivery, data-driven decision-making, and improved performance (Araz et al., 2020).” should be reorganized. “Further research is needed to explore how BDA can enhance healthcare supply chain management performance (HCSCMP) and to validate existing findings.” can be the last sentence and the later sentences can be integrated as drivers of HSCP.

  “RQ1. What is the number of academic studies on Big Data Analytics in the context of Supply Chain Management, and what research methods and data collection techniques have been used in these studies?”  This RQ can be shortened such as telling “investigating the trends in application of BDA in the HSCM”.

 “RQ2. How does the application of Big Data Analytics enhance efficiency in Healthcare Supply Chains Management according to existing studies?”. According to the abstract this study aims to investigate efficiency and performance of HSCM. But efficiency is only mentioned in the RQ2.

 The paragraph which is located after research questions should be significantly reorganized. I assume that the authors wanted to talk about the contribution of the study. However, it sounds more the findings or may be conclusion section.

**Comments for the Big Data and Big Data analytics in Healthcare Supply Chain Management**

 This section mainly talks about the big data. But it does not discuss big data in Healthcare Supply Chain Management.

**Comments for the Organizational Information Process Theory**

 Why did you specifically created a section titled as “Organizational Information Process Theory”?

**Comments for the** **Methodology**

 What is the reason of starting to screen in 2016?

 What are the keywords used for screening?

 Which journals (science category, index type etc.) were included?

 How did you analyze the papers included?

**Comments for the Results analysis**

 Please only use Figure 2 or Figure 3.

 “Research methods and data collection techniques have been used in big data analytics in the context of supply chain management studies”. Please add “health” before “supply chain management studies”.

 I did not understand to relating and discussing the country and research method (Table 1, Figure 5). Could you explain the importance of relating these two different findings?

**Comments for the Discussion**

 The discussion section seems the repetition of the results section. In order to overcome this problem, it would be better to synthesize and discuss the “Efficiency Enhancements of Big Data Analytics in Healthcare Supply Chains” and “Enablers and Challenges in Implementing Big Data Analytics in Healthcare Supply Chains” sections under one title.

**Reviewer Decision**

 I suggest the authors revise and develop the sections based on the comments.

Are the rationale for, and objectives of, the Systematic Review clearly stated?

Partly

Is the statistical analysis and its interpretation appropriate?

Partly

If this is a Living Systematic Review, is the ‘living’ method appropriate and is the search schedule clearly defined and justified? (‘Living Systematic Review’ or a variation of this term should be included in the title.)

Yes

Are sufficient details of the methods and analysis provided to allow replication by others?

Partly

Are the conclusions drawn adequately supported by the results presented in the review?

Yes

Reviewer Expertise:

Supply chain management; Logistics management

I confirm that I have read this submission and believe that I have an appropriate level of expertise to confirm that it is of an acceptable scientific standard, however I have significant reservations, as outlined above.

# Response

**Published:** 2024-11-14

**Authors:** دينا النعيمي

**Abstract:** Thank you for your valuable feedback. I have revised the abstract to clarify the study's aim, emphasizing that assessing the impact of BDA on performance, along with examining efficiency improvements and success factors, are interconnected objectives of the research. This should make the focus of the study clearer in the abstract.

**Location of Sentence on Descriptive Analysis and Content Analysis**:

 Thank you for this suggestion. We will reposition the sentence, *“The study began with a descriptive analysis of academic research on BDA in the context of SCM, followed by a content analysis to assess the impact of BDA-based management systems on HCSCMP and to identify the key enablers and challenges for implementing BDA in HCSC,”* to improve the flow of the introduction. This will help clarify the structure and methodology of the study within the introductory context.

 After research questions guide the study:

**Discussion of Structured and Unstructured Data** :

 We will revise the paragraph that starts with *“Adopting BDA in HCSCs facilitates real-time service delivery, data-driven decision-making, and improved performance,”* so that it concludes with, *“Further research is needed to explore how BDA can enhance healthcare supply chain management performance (HCSCMP) and to validate existing findings.”* This will allow the later sentences to flow naturally into a discussion of drivers in healthcare supply chain performance (HCSCP), following your helpful suggestion.

**Reorganizing the Paragraph on Adopting BDA**:

 We will revise the paragraph that starts with *“Adopting BDA in HCSCs facilitates real-time service delivery, data-driven decision-making, and improved performance,”* so that it concludes with, *“Further research is needed to explore how BDA can enhance healthcare supply chain management performance (HCSCMP) and to validate existing findings.”* This will allow the later sentences to flow naturally into a discussion of drivers in healthcare supply chain performance (HCSCP), following your helpful suggestion.

**Rephrasing Research Question 1** :

 RQ1 : What are the trends in the application of BDA in HCSCM?

**Clarifying Efficiency in RQ2** :

 Thank you for pointing out this discrepancy. We will modify the abstract to clarify that both efficiency and performance are central aims, ensuring consistency with RQ2’s focus on efficiency within healthcare supply chain management.

**Reorganizing the Paragraph Following the Research Questions**:

 We recognize that the paragraph after the research questions may seem more like conclusions or findings. To clarify, we will revise this section to focus on the study's intended contributions and will remove or relocate content that discusses specific findings.

**Comments for the Big Data and Big Data analytics in Healthcare Supply Chain Management**

 Section Revised

**Comments for the Organizational Information Process Theory**

 Thank you for your question. The 'Organizational Information Process Theory (OIPT)' section was included to provide a theoretical foundation for understanding how Big Data Analytics (BDA) supports healthcare supply chain management. OIPT explains how organizations can enhance their decision-making and operational efficiency by improving their information processing capabilities. Given that BDA is fundamentally about managing, analysing, and utilizing large volumes of data, OIPT offers a relevant lens to explore BDA’s role in healthcare supply chains. This theoretical framework helps explain how BDA can optimize information flow, reduce uncertainty, and support real-time decision-making, which are crucial for successful supply chain management in healthcare.

**Methodology:**

**Reason for Starting the Screening in 2016**:

 The screening period starts from 2016 because of a noticeable increase in academic research on Big Data Analytics in healthcare supply chains around that time. Starting in 2016 ensures the inclusion of recent and relevant studies, aligning with the growing interest and advancements in this area.

 The screening period begins in 2016 to capture recent developments and a noticeable rise in research on Big Data Analytics in healthcare supply chains during this period.

**Keywords Used for Screening**:

 Keywords likely included terms related to big data analytics, supply chain management, and healthcare, such as *“Big Data Analytics,” “Supply Chain Performance,” “Healthcare Supply Chain Management,”* and others relevant to enablers and challenges in BDA implementation.

 The keywords used included ‘Big Data Analytics,’ ‘Supply Chain Performance,’ ‘Healthcare Supply Chain Management,’ along with terms related to enablers and challenges in implementing BDA.

 Which journals (science category, index type etc.) were included?

 The review focused on peer-reviewed journals indexed in recognized databases like Scopus and Google Scholar, specifically in areas such as healthcare management, supply chain management, and information systems.

 We included peer-reviewed journals indexed in major databases like Scopus and Google Scholar, focusing on healthcare management, supply chain management, and information systems.

 How did you analyze the papers included?

 The analysis involved a systematic literature review methodology. The papers were screened, selected, and synthesized based on their contributions to understanding Big Data Analytics’ impact on healthcare supply chains, including descriptive and content analysis to identify key findings, enablers, and challenges.

 The included papers underwent descriptive and content analysis to synthesize insights on BDA’s impact on healthcare supply chains, focusing on efficiency improvements and identifying key enablers and challenges for successful implementation.

**Results analysis:**

**Comments for the Results analysis**

 Only use Figure 2 or Figure 3.

**Response to Reviewer:**

 Thank you for your feedback. I have revised the analysis to include only Figure 2, as suggested.

 “Research methods and data collection techniques have been used in big data analytics in the context of supply chain management studies”. Please add “health” before “supply chain management studies”.

 Additionally, I have updated the phrase to read, *‘Research methods and data collection techniques have been used in big data analytics in the context of healthcare supply chain management studies,’* to clarify the healthcare focus of this review."

 Discussing the country and research method (Table 1, Figure 5). Could you explain the importance of relating these two different findings?

 Thank you for highlighting this point. The purpose of relating countries with research methods in Table 1 and Figure 5 is to showcase regional preferences and methodological diversity in Big Data Analytics (BDA) research within healthcare supply chains. This comparison helps reveal how different regions prioritize certain research methods—such as questionnaires in North America and interviews in Australia—reflecting regional contexts, available resources, and academic traditions. By understanding these differences, we gain insight into the approaches that researchers in various regions use to investigate BDA’s impact on healthcare supply chain management, which could indicate unique challenges, cultural influences, or operational focuses in each region. This contextual understanding enhances the comprehensiveness of our review by demonstrating how geographic and methodological factors may shape research outcomes and perspectives on BDA implementation.

**Comments for the Discussion**

**Response to Reviewer:**

 Thank you for this helpful suggestion. To enhance the discussion and avoid redundancy, we will integrate the ‘Efficiency Enhancements of Big Data Analytics in Healthcare Supply Chains’ and ‘Enablers and Challenges in Implementing Big Data Analytics in Healthcare Supply Chains’ under a unified title, such as **‘Impact of Big Data Analytics on Healthcare Supply Chain Performance: Efficiency, Enablers, and Challenges.’** This synthesis will allow us to discuss both the benefits and obstacles of BDA implementation within a single framework, creating a more streamlined and interpretive analysis.

 By reorganizing this way, we can directly relate the efficiency improvements BDA offers to the specific enablers and challenges that influence its successful adoption. This will provide a more holistic view of BDA’s impact on healthcare supply chains, enabling readers to understand how these factors work together to enhance or inhibit supply chain performance.

# Reviewer report: Reviewer response for version 1

**DOI:** 10.5256/f1000research.171850.r333485

**Published:** 2024-11-09

**Authors:** Martin Beaulieu

**Affiliations:** 1 HEC Montréal, Montreal, Canada

On text form :

- Why do the authors combine the use of upper and lower case letters in the presentation of key words?
- The introduction starts very quickly, and the authors use acronyms without defining them or defining them further in the text (BDA or HCSCMP).
- I find it a little strange that figure 1 is in the introduction and not in the “Methodology” section.
- Is the name “CİĞERCİ” capitalized?

As far as the content of the paper is concerned, in the section “Big Data and Big Data analytics in Healthcare Supply Chain Management”, the authors tell us about Big Data, supply chain management, but they don't really deal with the Healthcare supply chain. What distinguishes the healthcare sector from other industries? See below comments.

 Why did you choose 2016 as the starting point for your reference search?

 I'm not convinced of the relevance of figure 3; it seems redundant with figure 2, or at the very least, I don't think it adds any real value.

 For the subsection answering question RQ2, is it possible to be more precise? There are two main supply chains in the healthcare sector: pharmaceuticals and medical supplies. Each presents its own challenges. So, the benefits suggested by these studies apply to which types of products? When I mentioned more development around HSCM in the literature review, the authors could expand on these ideas. This would give more depth to their discussion.

 I suggest that the authors consult these two references, as they may help the authors to develop some of the ideas in the literature review:

 Reference: (1,2)

Are the rationale for, and objectives of, the Systematic Review clearly stated?

Yes

Is the statistical analysis and its interpretation appropriate?

Partly

If this is a Living Systematic Review, is the ‘living’ method appropriate and is the search schedule clearly defined and justified? (‘Living Systematic Review’ or a variation of this term should be included in the title.)

Yes

Are sufficient details of the methods and analysis provided to allow replication by others?

Partly

Are the conclusions drawn adequately supported by the results presented in the review?

Partly

Reviewer Expertise:

healthcare logistics, supply chain management, inventory management, purchasing

I confirm that I have read this submission and believe that I have an appropriate level of expertise to confirm that it is of an acceptable scientific standard, however I have significant reservations, as outlined above.

## References

[1] : Digitalization of the healthcare supply chain: A roadmap to generate benefits and effectively support healthcare delivery. *Technological Forecasting and Social Change* .2021;167: 10.1016/j.techfore.2021.120717 10.1016/j.techfore.2021.120717

[2] : Prediction of heatwave related mortality magnitude, duration and frequency with climate variability and climate change information. *Stoch Environ Res Risk Assess* .2024;38(11) : 10.1007/s00477-024-02813-0 4471-4483 10.1007/s00477-024-02813-0 39492842 PMC11525403

# Response

**Published:** 2025-02-11

**Authors:** دينا النعيمي

**Reviewer Comment:**

- Why do the authors combine the use of upper and lower case letters in the presentation of key words?

**Author Response:**

 The inconsistency in using upper and lower-case letters in the keywords was unintended. This was standardized in the revised version, ensuring uniformity in formatting keywords to adhere to academic conventions.

**Reviewer Comment:**

- The introduction starts very quickly, and the authors use acronyms without defining them or defining them further in the text (BDA or HCSCMP).

**Author Response:**

 We acknowledge that acronyms such as BDA (Big Data Analytics) and HCSCMP (Healthcare Supply Chain Management Performance) were introduced without proper definition in the introduction. In the revised version, these acronyms defined upon first mention (IN THE Introduction Section).

**Reviewer Comment:**

  

- I find it a little strange that figure 1 is in the introduction and not in the “Methodology” section.

**Author Response:**

 Placement of Figure 1: The positioning of Figure 1 in the introduction section was intended to provide an early visual representation of the systematic review process. However, based on the feedback, this figure relocated to the “Methodology” section in the revised version, where it aligns more logically with the discussion of the research design.

**Reviewer Comment:**

  

- Is the name “CİĞERCİ” capitalized?

**Author Response:**

 Corrected:

**Correct Harvard-Style Formatting:**

**Ciğerci, M.** (2023) 'Main Effects of Big Data on Supply Chain Management. Implementation of Disruptive Technologies in Supply Chain Management,' *Yeditepe University Institute of Social Sciences*, Istanbul, Turkey, pp. 27–49.

**Correct In-Text Reference: **(Ciğerci, 2023).

**Reviewer Comment: **As far as the content of the paper is concerned, in the section “Big Data and Big Data analytics in Healthcare Supply Chain Management”, the authors tell us about Big Data, supply chain management, but they don't really deal with the Healthcare supply chain. What distinguishes the healthcare sector from other industries?

**Author Response:**

 In the revised version of the paper, we included the unique characteristics of healthcare supply chains (HCSCs) that differentiate them from other industries.

**Under the section titled "Big Data and Big Data Analytics in Healthcare Supply Chain Management".**

**2 ^nd^ paragraph:**

 HCSCs are fundamentally distinct from other industries due to their direct impact on human lives. The availability of medical supplies, medications, vaccines, and personal protective equipment (PPE) is critical for ensuring timely patient care, imposing higher demands for operational efficiency and resilience than conventional SCs (Govindan et al., 2022). HCSCs face significant challenges related to unpredictable demand, such as during pandemics like COVID-19, when the need for medications, ventilators, and vaccines surged unexpectedly, unlike traditional SCs that often experience steady demand patterns (Ivanov & Dolgui, 2021). Additionally, HCSCs are highly regulated to ensure patient safety and product quality, requiring compliance with stringent procurement, storage, and distribution standards, including temperature-controlled logistics for products like biologics and vaccines (Chen et al., 2021). The complexity of managing healthcare products, which often require specific handling conditions, adds unique logistical challenges (Ristevski & Chen, 2018). Moreover, BDA in HCSCs goes beyond improving operational efficiency to ensuring patient safety by predicting disease outbreaks, optimizing medication distribution, and reducing errors—functions rarely found in other industries (Nguyen et al., 2018). HCSCs prioritize patient-centred care, focusing on quality and reliability over cost savings, a priority that often distinguishes them from other sectors (Benzidia et al., 2023).

References (Added):

 Govindan, K., et al. (2022). Pandemic-induced disruptions in healthcare supply chains. *Supply Chain Management Review*, 28(2), 98-111.

 Ivanov, D., & Dolgui, A. (2021). A digital supply chain twin for managing disruptions. *International Journal of Production Research*, 59(14), 4180-4195.

**Reviewer Comment: **Why did you choose 2016 as the starting point for your reference search?

**Author Response:**

 Rationale for Selecting 2016 as the Starting Point: The selection of 2016 as the starting point for the reference search was deliberate. It aligns with the period when Big Data Analytics began gaining substantial traction in healthcare supply chain research, as highlighted by studies such as Nguyen et al. (2018) and Hofmann & Rutschmann (2018). This decision ensures the review captures recent advancements and remains relevant to contemporary developments in the field.

**Reviewer Comment: **I'm not convinced of the relevance of figure 3; it seems redundant with figure 2, or at the very least, I don't think it adds any real value.

**Author Response:**

 We agree with the reviewer’s observation. In the revised version, we removed Figure 3 to streamline the presentation of results and avoid redundancy.

 (The bar chart in Figure 3 illustrates the number of publications by different methods used in BDA research within the context of SCM. Each bar represents a distinct research method and the total number of publications employing that method. **Also removed**)

**Reviewer Comment: **For the subsection answering question RQ2, is it possible to be more precise? There are two main supply chains in the healthcare sector: pharmaceuticals and medical supplies. Each presents its own challenges. So, the benefits suggested by these studies apply to which types of products? When I mentioned more development around HSCM in the literature review, the authors could expand on these ideas. This would give more depth to their discussion.

**Author Response:**

 The BDA benefits suggested by the studies are pharmaceuticals, and medical supplies were included in the discussion section addressing RQ2.

**Included:**

 BDA significantly benefits the two main SCs in the healthcare sector: pharmaceuticals and medical supplies. BDA addresses challenges such as demand forecasting inaccuracies, drug shortages, and cold chain logistics for pharmaceuticals by leveraging predictive analytics to assess disease trends and optimize medication production and distribution. It also enhances real-time inventory monitoring to ensure compliance with strict storage requirements, especially for temperature-sensitive drugs like vaccines (Beaulieu & Bentahar, 2021; Ouarda et al., 2024). BDA mitigates high inventory variability and fluctuating demand in medical supplies by optimizing procurement processes through historical usage data and enabling agile supplier identification during emergencies, such as the COVID-19 pandemic. By providing end-to-end visibility across SCs, BDA reduces lead times and ensures timely order fulfilment, making it a critical tool for strengthening HCSC resilience and efficiency (Beaulieu & Bentahar, 2021).

 References (included):

 Beaulieu, M., & Bentahar, O. (2021). Digitalization of the healthcare supply chain: A roadmap to generate benefits and effectively support healthcare delivery. *Technological Forecasting and Social Change, 167*. https://doi.org/10.1016/j.techfore.2021.120657

 Ouarda, T. B. M. J., Masselot, P., Campagna, C., & Gosselin, P. (2024). Prediction of heatwave-related mortality magnitude, duration, and frequency with climate variability and climate change information. *Stochastic Environmental Research and Risk Assessment, 38*(11), 4471-4483. https://doi.org/10.1007/s00477-024-02179-5

**Reviewer Comment: **I suggest that the authors consult these two references.

**Author Response:**

 Yes, Suggested references included in the discussion section.
